# Liquid-liquid phase separation in cell physiology and cancer biology: recent advances and therapeutic implications

**DOI:** 10.3389/fonc.2025.1540427

**Published:** 2025-03-31

**Authors:** Ziyuan Huang, Zimeng Liu, Lieqian Chen, Yanlin Liu, Gaofei Yan, Yizheng Ni, Qiuxia Yan, Wenqian He, Junhong Liu, Shufang Luo, Jindong Xie

**Affiliations:** ^1^ Department of Urology, The First Huizhou Affiliated Hospital of Guangdong Medical University, Huizhou, China; ^2^ Computational Medicine and Epidemiology Laboratory (CMEL), Guangdong Medical University, Zhanjiang, China; ^3^ School of Medicine, Sun Yat-Sen University, Shenzhen, China; ^4^ Department of Clinical Medicine, Hunan University of Medicine, Huaihua, Hunan, China; ^5^ Department of Critical Care Medicine, The Fifth Affiliated Hospital, Southern Medical University, Guangzhou, China

**Keywords:** liquid-liquid phase separation, cancer molecular mechanism, cancer biology, cancer therapy, pathophysiology

## Abstract

Liquid-liquid phase separation (LLPS) is a pivotal biophysical phenomenon that plays a critical role in cellular organization and has garnered significant attention in the fields of molecular mechanism and pathophysiology of cancer. This dynamic process involves the spontaneous segregation of biomolecules, primarily proteins and nucleic acids, into condensed, liquid-like droplets under specific conditions. LLPS drives the formation of biomolecular condensates, which are crucial for various cellular functions. Increasing evidences link alterations in LLPS to the onset and progression of various diseases, particularly cancer. This review explores the diverse roles of LLPS in cancer, highlighting its underlying molecular mechanisms and far-reaching implications. We examine how dysregulated LLPS contributes to cancer development by influencing key processes such as genomic instability, metabolism, and immune evasion. Furthermore, we discuss emerging therapeutic strategies aimed at modulating LLPS, underscoring their potential to revolutionize cancer treatment.

## 1 Introduction

**Figure 1:**
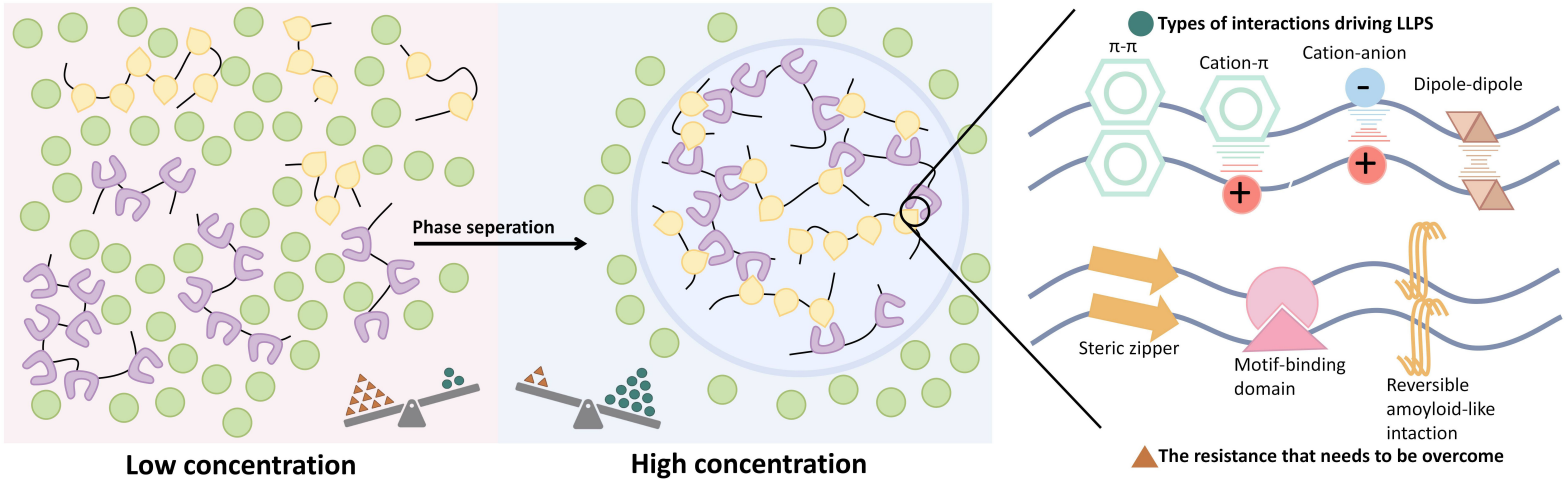
The basic process of LLPS and some types of interactions driving LLPS. LLPS refers to the process by which biomolecules, such as proteins and nucleic acids, undergo transient, weak interactions to form enriched liquid-like microenvironments within cells, resulting in two distinct liquid phases separated from the surrounding environment, enabling membrane-less compartmentalization.

Liquid-liquid phase separation (LLPS) is a physicochemical process in which biomolecules separate into distinct two liquid phases (1), forming membrane-less biomolecular condensates enriched with specific proteins and nucleic acids. Unlike other phase separation forms, such as solid-liquid, LLPS is dynamic and reversible, enabling the spatial organization of cellular components (2). Through various intermolecular forces, macromolecules segregate into dense and dilute phases, resulting in condensates that concentrate specific biomolecules in liquid-like regions while excluding others (**Figure 1**) (3–5). The intrinsically disordered region (IDR) of proteins primarily mediates aggregation through electrostatic interactions, facilitating their LLPS (6). Nucleic acids, particularly RNA, can promote LLPS by interacting electrostatically with protein IDRs and can also be incorporated into the membrane-less complexes formed by these proteins (7). Additionally, nucleic acids themselves can also drive LLPS, with nucleotide repeat sequences likely playing a key role in mediating this process (8). Furthermore, van der Waals forces and hydrogen bonding provide supplementary intermolecular interactions that strengthen transient binding and facilitate the formation of networks among biomolecules, thereby stabilizing LLPS droplets (9). These biomolecular condensates include entities such as stress granule (SG), transcriptional regulatory complexes, and synaptic densities, all of which are essential for maintaining cellular homeostasis and facilitating responses to environmental changes (9–12).

Cancer, a complex group of diseases, is influenced by various factors. Recent studies have revealed intricate connections between LLPS and the hallmarks of cancer. The formation and dysregulation of condensates mediated by LLPS not only impact the gene expression of cancer cells (13) but also their metabolism (14), proliferation (15), invasiveness (16), playing key roles in tumor initiation and progression. Furthermore, LLPS influences the tumor microenvironment and the interactions between cancer cells and their surroundings by regulating immune responses (17). Therefore, understanding the LLPS process, particularly its role in cancer characteristics, is essential for developing novel targeted anti-cancer strategies.

Although LLPS has been extensively studied as a cellular biology phenomenon, its exact mechanism is still difficult to fully elucidate. This review aims to synthesize current findings, explore the critical roles of LLPS in molecular mechanism and pathophysiology of cancer, and analyze its potential implications in cancer development and progression. Additionally, we discuss potential therapeutic targets for dysregulated LLPS, offering new perspectives for future research directions and clinical applications. Current research is uncovering connections between LLPS and cell fate determination, including cell cycle regulation, cell death induction, and differentiation. This review aims to synthesize current research findings, explore the critical roles of LLPS in cell physiology and cancer biology, analyze its potential implications in cancer development and progression. And discuss potential therapeutic targets for dysregulated LLPS, providing new perspectives for future research directions and clinical applications.

## 2 Fundamental principles and regulatory mechanisms of LLPS

### 2.1 Related regions for LLPS occurrence in biomolecules

#### 2.1.1 IDRs play a key role in LLPS

The LLPS of proteins containing IDRs has been proposed as a mechanism for the formation of membrane-less organelles (18). Unlike structured protein domains, IDRs lack stable secondary or tertiary structures and often exhibit low complexity domain (LCD) of repetitive sequence elements, providing the basis for intermolecular interactions (6, 18). Studies have confirmed that LLPS is promoted by one or more regions with high negative charge density, along with aromatic and hydrophobic residues distributed throughout the sequence. These interactions depend on specific amino acid residues or sequences within IDRs, regardless of their exact sequence. Specifically, aromatic (tyrosine, tryptophan) and hydrophobic (leucine, methionine) residues in IDRs are sufficient to drive LLPS through charge interactions (19).

Although protein-nucleic acid LLPS mediated by IDRs is traditionally thought to involve non-specific electrostatic and hydrophobic interactions, recent research has revealed that specific residues within IDRs can drive novel mechanisms of LLPS. Dang et al. (20) discovered that IDRs containing Arg/Lys residues serve as unique binding domains for ATP and nucleic acids. Single stranded DNA (ssDNA) binds to Arg and Lys residues with unique affinity, unlike traditional non-specific electrostatic and hydrophobic interactions. Moreover, ssDNA can both promote and dissolve LLPS, with the length of ssDNA—rather than their sequence—determining their effect. High cellular concentrations of ATP can regulate LLPS by competitively binding to Arg/Lys residues. These findings suggest that IDRs mediate LLPS through multiple mechanisms.

#### 2.1.2 Other structural domains involved in LLPS

Other structural domains may also contribute to LLPS formation, including stable protein structures that contrast with IDRs, as summarized in **Table 1**.

**Table 1:** Other structural domains involved in LLPS and their functional implications.

Domain Name	Composition of Condensate	Interaction	Condensate Effect	Reference
SH2	SHP2-FGFR2-PLCγ1	Multivalent interaction	As a switch of enzyme activity in RTK mediated pathway	(21)
PrLDs	FUS	Multivalent interaction	Mediating the occurrence of neurodegenerative diseases	(22)
Trimeric domain	EML4-ALK	Multivalent interaction	Activation of the RAS/MAPK signaling pathway	(23)
Ahpc-TSA	PRDX1-IncRNA	Multivalent interaction	Accumulation of lipid peroxidation via the SLC7A11-GPX4 axis, which disrupts intracellular ROS homeostasis	(24)
HOXA9 homologous domain	NHA9-DNA	Additional heterotypic interactions	Driving the formation of WBC	(25)
PTP	SHP2	Multivalent interaction	Activation of the RAS/MAPK signaling pathway	(26)
SAM	Liprin-α1	Short linear interaction motif	Assembling large higher-order molecular complexes	(27)

SH2, Src homology 2; FGFR2, fibroblast growth factor receptor 2; SHP2, SH2 domain-containing protein tyrosine phosphatase 2; PLCγ1, 1-phosphatidylinositol 4,5-bisphosphate phosphodiesterase gamma 1; PrLDs, prion-like domains; PRDX1, peroxiredoxin1; IncRNA, long non-coding RNA; HOXA9, homeobox A9; NHA9: NUP98–HOXA9; WBC, white blood cell; PTP, protein tyrosine phosphatase; SAM, Sterile Alpha Motif.

### 2.2 Regulatory mechanisms of LLPS

**Figure 2:**
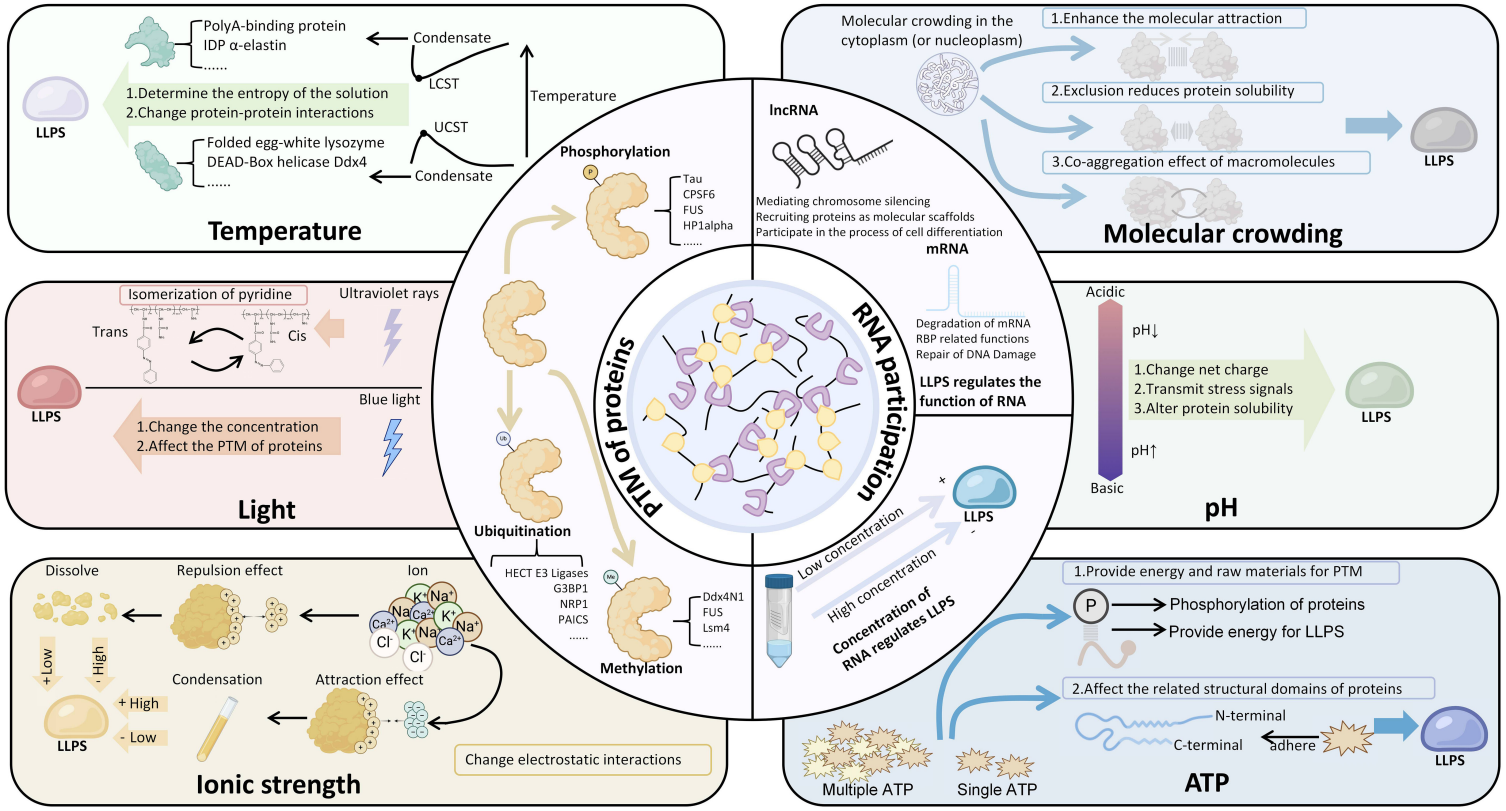
The regulatory mechanisms of LLPS. Among them, the concentration of RNA can regulate LLPS, and LLPS can also regulate the physiological functions of related RNA. Many environmental factors manifest as bidirectional regulation of LLPS, such as pH, ATP, temperature, ionic strength, etc. The figure also summarizes the condensates involved in the regulation of relevant factors. LCST, Lower Critical Solution Temperature; UCST, Upper Critical Solution Temperature; PTM, Post-Translational Modifications (PTM).

The regulatory mechanisms of LLPS are complex and diverse, involving post-translational modification (PTM) of proteins, RNA participation, and changes in environmental conditions (**Figure 2**).

#### 2.2.1 PTMs of proteins

PTMs of proteins, such as phosphorylation (28–31), ubiquitination (32–35), acetylation (18), and methylation (36), play a crucial role in LLPS. PTMs primarily promote condensate formation by altering the LLPS threshold of proteins containing IDRs. Due to the high flexibility and inherent instability of IDRs, PTMs can significantly modify their charge and hydrophobic interactions, thereby regulating the dynamics and stability of LLPS (11, 37). In a study by Yang et al. (7), it was demonstrated that phosphorylation can modulate electrostatic interactions between three IDRs, influencing LLPS. Specifically, fine-tuning the phosphorylation of IDR1 in G3BP1 increases its negative charge, which, by interacting with the positively charged IDR3, promotes the precipitation of G3BP1 and RNA condensates. This finding confirms that condensate formation is driven by changes in charge rather than other effects of IDR residues. Methylation, particularly arginine methylation, can alter the shape and charge distribution of proteins, increase hydrophobicity, and reduce their hydrogen bond donor capacity. These changes are crucial for intermolecular interactions, particularly for π-cation interactions (38). Wang et al.’s study (39) confirmed that multivalent interactions mediated by arginine methylation can create additional binding sites for Tudor domain-containing proteins, such as survival of motor neuron (SMN) proteins, thereby lowering the threshold for LLPS in protein-RNA complexes. Another study demonstrated that acetylation/deacetylation of IDRs can regulate LLPS. The N-terminal IDR of RNA helicase DDX3X undergoes LLPS *in vitro*, and acetylation at multiple lysine residues impairs droplet formation. This impairment is linked to the neutralization of the positively charged lysine by acetylation, which disrupts the intermolecular interactions between cationic lysine and anionic or aromatic π residues. The deacetylase HDAC6 can remove acetyl groups from DDX3X-IDR1, enhancing its tendency to undergo LLPS. Therefore, deacetylase inhibitors may play a crucial role in modulating LLPS (18). These findings collectively confirm that PTMs can participate in the LLPS process by modifying intermolecular interactions.

#### 2.2.2 RNA participation

RNA not only plays a structural role in condensate assembly but also has regulatory functions that help maintain the liquid-like states of biomolecular condensates. The interaction between RNA and proteins is a key process in LLPS, where the protein interaction network functions as a complex (node) of interconnected RNA binding domain (RBD). The combined RNA binding ability of these domains determines whether LLPS occurs during RNA influx (40). RNA mediates LLPS through unique RBD-RNA interactions and acts as a scaffold molecule to promote LLPS, stabilizing various prototype cell cohesive collectives including nucleosomes, cajal bodies, SGs, and P-bodies (41). SGs, as an example of membrane-less ribonucleoprotein assembly, emerge under a series of environmental pressures. These dynamic condensates are storage repositories for a large number of proteins, including but not limited to TIA-1, TTP, and G3BP (42), as well as transient translation silenced mRNA molecules (43). Among them, G3BP1 serves as a key molecular switch that can trigger RNA dependent LLPS in response to an increase in intracellular free RNA concentration. The RBD of G3BP1 consists of two different RNA binding entities, RRM domain and IDR3, which can independently promote SGs assembly and participate in the stress response of various cancer cells, including sarcomas, to the outside world (7, 44). Meanwhile, the interaction between IDR3 and two ribosomal proteins (RPS6 and RPS23) also participates in the assembly of SGs (45). In particular, a study demonstrated that RNA critically regulates the phase behavior of prion-like RNA-binding protein (RBP). They found that low RNA/protein ratios promote phase separation into liquid droplets, while high RNA/protein ratios inhibit droplet formation, thereby highlighting the role of RNA in maintaining the dynamic properties of these condensates (46). In another study, it was confirmed that the heterologous interaction between nuclear proteins and rRNA can also drive LLPS and participate in the division and LLPS of nucleosome scaffold proteins (41).

Moreover, researchers highlighted that enhancer RNA (eRNA) can modulate the physicochemical properties of transcriptional condensates, promoting a more dynamic liquid-like state that facilitates enhancer activation and chromosomal reorganization (47). The structural and sequential characteristics of RNA are pivotal in modulating LLPS. Studied have elucidated that the secondary structure of mRNA exerts a significant influence on the aggregation dynamics of polyglutamine (polyQ) proteins through the regulation of LLPS. More precisely, distinct mRNA sequences and their corresponding secondary structures exhibit differential regulatory impacts on the LLPS behavior of polyQ proteins. Notably, specific mRNA configurations, such as hairpin structures, have been shown to markedly potentiate the LLPS of polyQ proteins. This enhancement is attributed to the fact that the secondary structure of mRNA dictates the intensity of its multivalent interactions with polyQ proteins, thereby influencing the LLPS process (48).

The pronounced propensity for GC base pairing enables RNA molecules containing CAG and CUG trinucleotide repeats to autonomously self-assemble into liquid-like condensates, a phenomenon observed both *in vitro* and *in vivo* (8). Similarly, RNAs with well-defined structural motifs, such as G-quadruplexes, have been demonstrated to undergo aggregation under *in vitro* conditions in the presence of molecular crowding agents (49). These findings underscore the critical role of RNA sequence specificity and higher-order structural conformations in the regulation of condensation processes.

#### 2.2.3 Environmental conditions

Environmental conditions profoundly influence LLPS, with emerging evidence revealing complex mechanisms of condensate formation and regulation. Recent studies have demonstrated that cellular stress triggers significant changes in biomolecular condensates (50, 51). For instance, a study showed that hyperosmotic stress induces YAP protein to form liquid-like nuclear condensates within seconds, reorganizing genome structure and activating specific gene expression programs (50). Similarly, researchers discovered that the ubiquitin-like modifier Urm1 facilitates stress-dependent phase separation, enabling cellular stress resilience (52). Urm1 modification enhances the phase separation of proteins under stress, acting as a reversible molecular “adhesive” that drives protective condensate formation. Changes in pH can alter the charge states of proteins and RNA molecules, thereby affecting their intermolecular interactions and modulating LLPS (53, 54). Temperature changes impact molecular thermal motion, with increased temperatures generally promoting the formation of transient, liquid-like assemblies that facilitate LLPS (55–57). Moreover, the degree of molecular crowding within cells is another crucial factor; high concentrations of macromolecules typically promote LLPS, while low concentrations may be insufficient to induce it (58, 59).

The specific mechanisms mediating LLPS can vary depending on the cellular environment. Researchers found that LLPS is driven by the cooperation of electrostatic and hydrophobic interactions at low salt concentrations, while the same process at high salt concentrations is primarily favored by hydrophobic and non-ionic interactions (60). Other studies have also confirmed that ion strength has an impact on LLPS (61, 62). Additionally, the role of hormones, such as estrogen and androgens, has been highlighted in recent literature, demonstrating that they can trigger the assembly of transcriptional condensates through phase separation mechanisms, which is particularly relevant in the context of cancer. For example, the androgen receptor (AR) forms liquid-like condensates in prostate cancer cells upon androgen stimulation, which correlates with transcriptional activity and oncogenic programs (51). These findings not only expand the traditional understanding of LLPS but also underscore the importance of environmental conditions in regulating this dynamic process. While environmental factors such as ATP levels (63) can modulate LLPS dynamics by altering intermolecular interactions, the role of light exposure in LLPS has primarily been studied in artificial systems. For example, *in vitro* LLPS experiments and optogenetic approaches have been used to cluster IDRs and induce LLPS in a controlled manner (64, 65). However, these systems rely on light-dependent moieties derived from plants, which do not inherently possess the capacity for LLPS without the presence of IDRs. Therefore, caution is warranted when interpreting light as a natural environmental regulator of LLPS, as current evidence is largely derived from engineered experimental setups rather than naturally occurring biological contexts.

Understanding these fundamental principles and regulatory mechanisms is crucial for elucidating the roles of LLPS in both physiological and pathological cellular states. Accumulating evidence highlights the critical involvement of LLPS in various cellular processes, including gene expression regulation, signal transduction, and stress response (66–68). Conversely, dysregulated LLPS has been closely linked to the development and progression of various diseases, particularly cancer. Ongoing research continues to illuminate the multifaceted functions of LLPS in cancers, offering insights that could inform targeted therapeutic strategies for LLPS-related cancers.

## 3 Roles of LLPS in molecular mechanism and pathophysiology of cancer

### 3.1 Associated condensates in cancer

Accumulating evidence from related studies has demonstrated that biomolecular condensates formed through LLPS, and their associated regulatory effects play a critical role in the initiation and progression of cancer. In this review, we summarize the key areas of some condensate and its role in cancer in **Table 2**. These condensates dynamically organize key signaling molecules and oncogenic factors, contributing to the dysregulation of cellular processes and driving tumorigenesis and cancer progression.

**Table 2:** Cancer associated condensates and their main functions.

Cancer	Related Condensate	Key Areas/Areas	Mechanism	Reference
Lung Cancer	EZH2	N-terminal glycine of EZH2	EZH2 undergoes LLPS to form condensates that sequester STAT3 and activates STAT3 signaling.	(69)
	EML4-ALK	N-terminal trimeric domain of EML4	LLPS forms a unique subcellular platform for tissue carcinogenic RTK and RAS signaling.	(23)
Breast Cancer	PRDX1	Ahpc-TSA domain of PRDX1	LLPS dysregulation of PRDX disrupts intracellular ROS homeostasis through the SLC7A11-GPX4 axis.	(24)
	AKAP95	101-210 of AKAP95	LLPS regulation of AKAP95 transcription process and RNA splicing.	(70)
Colorectal Cancer	NOP53	IDR1 domain of NOP53	The LLPS of NOP53 negatively regulates the p53 pathway and enhances tumor radiation tolerance.	(71)
Prostatic Cancer	AR	Unordered N-terminal domain of AR	The LLPS behavior of AR mediates its transcriptional activity and participates in the resistance to androgen drugs.	(72)
	Rbm14	C-terminal of Rbm14	RBM14 maintains prostate specific antigen expression through LLPS.	(17)
Liver Cancer	Glycogen		Glycogen LLPS can trigger the assembly of Laforin-Mst1/2 complex in glycogen droplets and inactivate Mst1/2, thereby activating Yap to promote the survival and growth of cancer cells.	(73)
Ewing Sarcoma	EWS-FLI	Prion-like Domain of EWSRI	LLPS of EWS-FLI can activate abnormal transcriptional programs and recruit BAF complexes to induce activation of target oncogenes.	(74, 75)
Pancreatic Cancer	KMT2D	LCDs of KMT2D	KMT2D LCDs can induce changes in cell proliferation and metastasis pathways in a H3K4me1 dependent manner by activating LIFR and KLF4.	(76)
Leukemia	PML-NBs	N-terminal domain of PML	Disrupting the LLPS process of PML-NBs can cause delayed recruitment of DNA repair proteins and disrupt gene stability.	(6, 77, 78)
	NHA9	C-terminal HOXA9 homologous domain of NHA9	LLPS drives the formation of NHA9 puncta in cells, promoting the transformation and expression of abnormal *Hox*.	(25)
	YY1	Histidine cluster in the YY1 activation domain	YY1 binds to HDAC1/3 and regulates METTL3 expression through moderate LLPS.	(79, 80)
	YTHDC1-m6A	Glu-rich N-terminal IDR and Arg/Pro-rich C-terminal IDR of YTHDC1	The nuclear condensate formed by the binding of YTHDC1 and m6A can control the survival and differentiation of AML cells.	(81)
Multiple Types of Cancer	SHP2	PTP domain of SHP2.	The LLPS of SHP2 mutant enhances the phosphorylation levels of MEK1/2 and ERK1/2, leading to excessive activation of RAS-MAPK.	(26, 82)
	MRNIP	IDR1 domain of MRNIP	MRNIP condensate can induce autophosphorylation of ATM and activate DNA damage response signals.	(83, 84)
	53BP1	IDR domain of 53BP1	The LLPS process of 53BP1 can promote DNA double stranded repair and enhance the function of the tumor suppressor gene p53.	(84–87)

### 3.2 The role of LLPS in molecular mechanism of cancer

#### 3.2.1 LLPS affects signaling transduction

**Figure 3:**
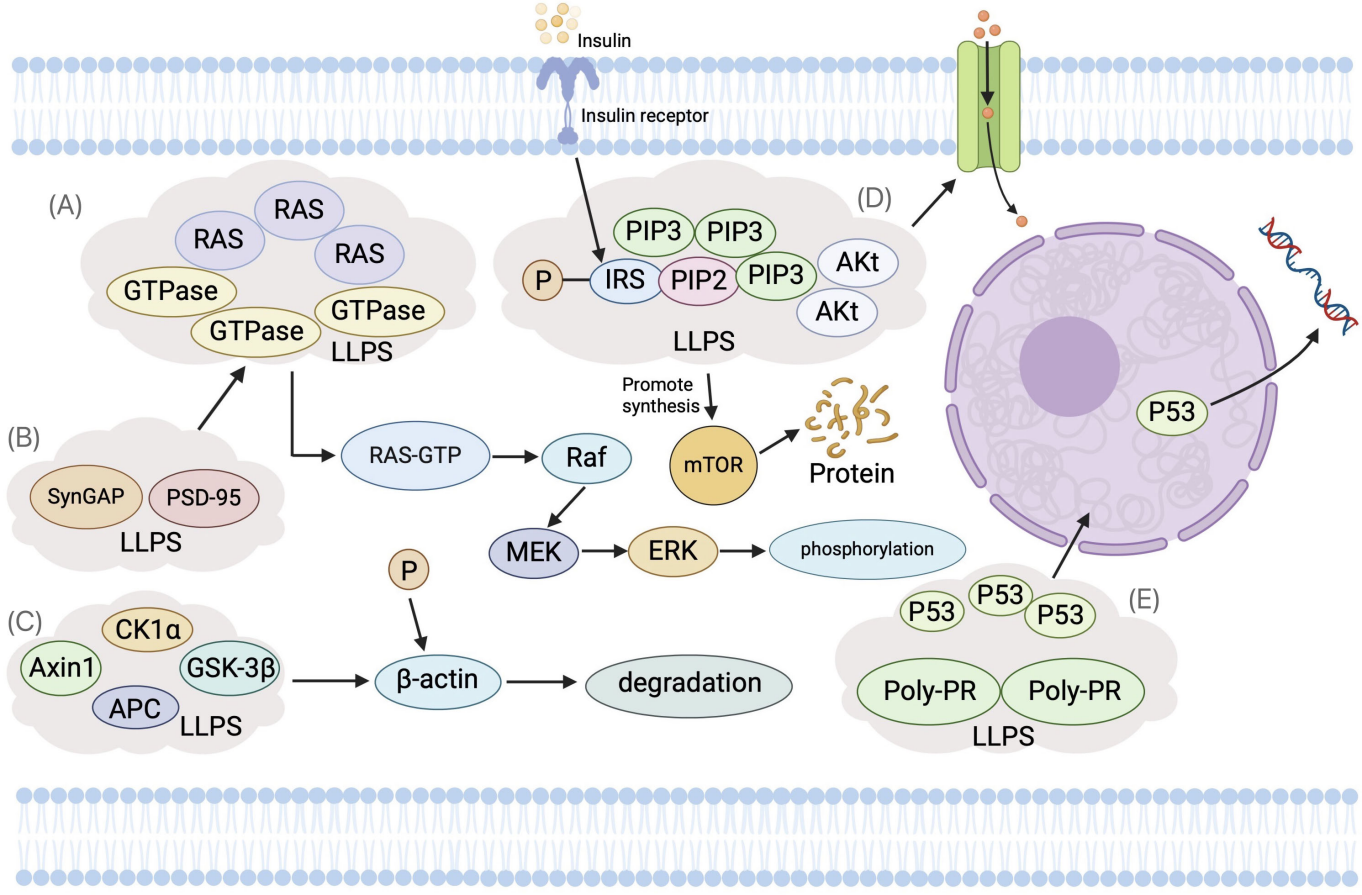
The Role of Liquid-Liquid Phase Separation (LLPS) in Cellular Signal Transduction Pathways. **(A)** Ras Signaling Pathway: Ras proteins, as small GTP-binding entities, utilize LLPS to form phase-separated microenvironments near the cell membrane. This increases the local concentration of Ras and its effectors, enhancing signal transduction. Raf kinase forms LLPS droplets with scaffold protein KSR1, enriching downstream kinases MEK and ERK. **(B)** SynGAP and PSD-95: These proteins experience concentration-dependent LLPS, creating dynamic, membrane-deficient, protein-rich phases. This hinders Ras/Rap GTPase activation, thus modulating the Ras signaling pathway. **(C)** Wnt Signaling Pathway: LLPS facilitates the assembly of the β-catenin destruction complex (DC), wherein Axin1 and APC (Adenomatous Polyposis Co) form condensates concentrating other DC components. This promotes β-catenin phosphorylation and degradation. Upon Wnt pathway activation, LRP6 phosphorylation inhibits Axin1’s phase separation, reducing DC assembly. DC condensates interact with Wnt receptor complexes, forming larger aggregates crucial for signal transduction. **(D)** Insulin Signaling Pathway: Upon insulin stimulation, PI3K and its effectors, like Akt, localize to specific membrane regions, forming phase-separated condensates. This spatial organization amplifies PI3K activity, providing efficiency and specificity in signal transduction through more effective interactions with substrates and regulatory partners. **(E)** Tumor Suppressor p53: The transactivation domain of p53 binds to Poly-PR or Poly-GR sequences, inducing LLPS, which affects transcription regulation, cell cycle control, DNA repair, and apoptosis.

LLPS dynamically compartmentalizes key components involved in signal transduction, creating distinct microenvironments within the cytoplasm or nucleus. These LLPS-driven microdomains significantly enhance the efficiency and precision of signaling pathway regulation (88–92) (**Figure 3**). Notably, such spatial organization plays a pivotal role in cancer initiation and progression, as it facilitates the aberrant activation or suppression of oncogenic signaling cascades. In the signaling pathways involved in the figure, the disease-related mutation SHP2 can undergo LLPS and recruit condensates, leading to overactivation and dysregulation of RAS-MAPK signaling, which is crucial for tumorigenesis events (26, 93). As a key component of the Wnt pathway, β-catenin induces aberrant LLPS through IDR-IDR interactions to promote the overactivation of Wnt signaling, which is one of the early events leading to cancer (67, 94). The transactivation domain of p53 can bind to Poly PR or Poly GR sequences to induce LLPS, thereby affecting transcriptional regulation, cell cycle control, DNA repair, and apoptosis (91).

#### 3.2.2 LLPS is involved in epigenetic changes

Epigenetics refers to phenotype changes that occur independently of genotype changes, and its modifications include DNA methylation, histone modifications (methylation and acetylation), and functional abnormalities in non-coding RNAs (95). Recent studies have demonstrated a significant interplay between LLPS and epigenetic modifications. Among these modifications, histone alterations play a pivotal role. In Drosophila melanogaster, LLPS has been implicated in the biogenesis of histone locus bodies (HLBs), which play a crucial role in the transcriptional regulation and post-transcriptional processing of histone mRNAs. This process directly influences histone biosynthesis and their subsequent modifications, thereby modulating chromatin dynamics and gene expression (96). Histone acetylation, particularly of histone 1 (H1), neutralizes the positive charges on histone tails, resulting in chromatin decondensation and subsequent genome activation, which may be the reason for the genomic instability of tumor cells. Notably, the length of linker DNA between nucleosomes has been identified as a critical regulatory factor in chromatin LLPS (97). Building on the role of H1, studies have demonstrated that this linker histone can partition DNA into liquid-like condensates, thereby acting as a scaffold for LLPS-mediated heterochromatin domain formation (98–101). This phenomenon has been experimentally validated in HeLa cells, where histone H1 undergoes condensation into liquid-like droplets within the nucleus. These droplets function as scaffolds to spatially segregate heterochromatin domains from DNA, thereby modulating the landscape of specific histone modifications in eukaryotic cells (98). Other histone modifications, such as H3K27me3, also play critical roles in gene expression regulation through LLPS. The interaction between H3K27me3 and poly-comb repressive complex 1 (PRC1) compresses nucleosomes in heterochromatin regions, inducing droplet formation and maintaining a repressive chromatin state (102); In another study, the LLPS of the H3K27me3 reader Bromo-adjacent homology-plant homeodomain containing protein 1 (BP1) is involved in transcriptional inhibition, highlighting LLPS’s importance in gene expression regulation (103).

Li et al. (76) found that histone lysine methyltransferase 2D (KMT2D) has two different LCDs, which can drive the assembly of membrane free condensates, thereby promoting the catalysis of H3K4, and promoting the occurrence of pancreatic cancer. LLPS can also locally regulate chromatin status and induce carcinogenesis by enriching key epigenetic modification enzymes, such as histone methyltransferase and acetyltransferase.

#### 3.2.3 Mutations in IDRs of cancer-associated proteins

IDRs, as phase separated molecular switches, finely regulate the function of cancer-related proteins through their sequence features and PTMs. LLPS has emerged as a fundamental mechanism underlying the establishment of dysregulated gene expression programs through the spatial organization of gene products. Notably, transcription factors can utilize their IDRs, particularly those enriched in activation domains, to initiate LLPS and subsequently regulate gene activation processes. This phenomenon has been extensively characterized in the context of the transcriptional coactivator MED1, where its IDRs facilitate the selective binding and concentration of the pluripotency factor OCT4. Mechanistically, this process is mediated by specific electrostatic interactions predominantly contributed by acidic amino acid residues within MED1’s IDRs. Such molecular interactions not only promote the formation of functional transcriptional enhancer complexes but also significantly amplify OCT4-mediated gene expression (67). Importantly, this LLPS-driven regulatory mechanism may provide a molecular basis for the observed overexpression of OCT4 in cancer stem cells (CSCs), potentially contributing to the maintenance of stemness properties and tumorigenic potential in malignant cells (104). This has also been confirmed in other studies, such as the fusion of LLPS from PHR/CRY2 constructs with TAF15 (105) or VP16 into IDRs like FUSN (106), which can amplify gene expression and increase transcriptional activation.

Recent studies have provided compelling evidence that tumor fusion proteins frequently exploit IDRs to dysregulate LLPS processes, with this phenomenon being particularly prevalent in hematological malignancies. A prominent example is the NUP98 fusion proteins (e.g., NUP98-HOXA9 and NUP98-PSIP1), where phenylalanine-glycine-rich repeat-containing IDRs facilitate the formation of nuclear condensates. These aberrant condensates have been shown to play a crucial role in promoting the self-renewal capacity of leukemia stem cells, thereby driving leukemogenesis (107). Similarly, in EWS, the fusion protein’s transcriptional activation domain, which comprises an extensive IDR spanning approximately 280 amino acid residues, demonstrates LLPS-dependent oncogenic activity. Mechanistic studies reveal that specific tyrosine residues within this domain mediate critical electrostatic interactions that govern condensate formation (108). These findings collectively underscore the pathological significance of IDR-mediated LLPS in cancer, particularly in the context of fusion oncoproteins, and highlight potential therapeutic targets for intervention in both hematological and solid tumors.

### 3.3 LLPS in cancer physiology

#### 3.3.1 Regulation of genome instability in cancer cells

The maintenance of genomic integrity largely relies on the DNA damage response (DDR) pathway, and the expression of key proteins in the DDR process can affect the regulation of tumor genomic instability (109). The formation of relevant condensates is involved in DDR reactions. For example, reducing the expression of Sentrin/SUMO specific protease 1 (SENP1) can improve DDR efficiency and cancer cells’ resistance to DNA damaging agents, which is related to SENP1 preventing RNF168 from forming nuclear condensate and confirming that SENP1 is a potential target for cancer therapy (110). DDR to DNA double strand breaks (DSBs) are triggered by the recognition of exposed DNA ends by the MRE11-RAD50-NBS1 (MRN) sensor complex. When DSBs occur, MRN interacting proteins (MRNIP) can form liquid like condensates, recruit and concentrate MRN complexes, rapidly mobilize them to damaged DNA sites, which in turn induce autophosphorylation of ATM and activate key kinases in the DNA damage response signaling cascade (83, 111).

P53 binding protein 1 (53BP1) plays an important role in DDR and maintaining genomic stability (112). Emerging research has confirmed that 53BP1 can both promote the formation of DDR clots through LLPS and recruit platforms for p53 and its co-activators when encountering DNA damage, thereby stabilizing p53 and promoting its function, and this is due to the assembly of transcription promoters induced by DSB, which drives RNA synthesis and stimulates the LLPS of DDR factors in focal form (84–87). It is worth noting that a study has shown that LLPS of 53BP1 can also maintain heterochromatin integrity and genomic stability independently of the DDR process, but has significant deficiencies in inhibiting heterochromatin transcription, indicating that 53BP1 condensate has multiple pathways in maintaining genomic stability (86). Fused in sarcoma (FUS), as an important RNA binding protein, can also form liquid compartments in the cytoplasm at DNA damage sites and under pressure (113), and mediate the recruitment of various DDR molecules, including 53BP1, at DNA damage sites, thereby mediating early responses to DDR (114).

In a separate investigation, researchers identified that ciRS-7, a circRNA harboring more than 70 potential miRNA-induced silencing complex (miRISC) binding sites, significantly promotes the LLPS of miRISC. Interestingly, ciRS-7 also promotes radiation-induced DNA repair, suggesting that ciRS-7-induced mi-RISC condensates may further facilitate DSB repair (115).

In other studies, it has been confirmed that environmental factors that alter LLPS may regulate DDR, such as the influence of salt concentration and pH on the LLPS process of Y14 and RNA *in vitro* (116). Changing these external conditions may affect the genomic stability in cancer cells. However, there is currently no direct literature to support and further exploration is needed. In summary, the formation and dissolution of condensates mediated by LLPS provide a highly dynamic and adaptive platform for cellular genomes, enabling them to quickly and accurately respond to internal and external environmental changes.

#### 3.3.2 Sustaining proliferation and resistance to cell death

The sustained proliferation and resistance to apoptosis in cancer cells are among their most prominent biological features, and these processes are closely linked to LLPS. LLPS can affect the condensation status and activity of key proteins in cell cycle regulation. In cells, hexokinase (HK) competes with Bax to bind to Voltage- Dependent Anion Channel I (VDAC1) on the mitochondrial membrane. Tau-441 condensate can recruit HK, leading to a decrease in the amount of free HK in the cytoplasm and an increase in the chance of Bax binding to VDAC1, resulting in an increase in Bax mediated apoptosis (117). Liu et al. (15) found that CPSF6 can regulate alternative polyadenylation and cancer cell proliferation through LLPS: The reduction of CPSF6 LLPS, rather than changes in its expression level, leads to a 3 ‘shortened UTR of cell cycle related genes and accelerates cell proliferation. TAZ condensate is in the nucleus under physiological conditions, while YAP condensate is in both the cytoplasm and nucleus, both of which can respond to the Hippo pathway (118, 119). Among them, nuclear TAZ condensate can not only promote the segmentation of other transcription factors, including its DNA binding partner TEAD, transcriptional co activator CDK9, BRD4, MED1 and active RNA polymerase II, but also promote the expression of downstream target genes, such as CTGF and CYR61, thus promoting the infinite proliferation signal of Hippo pathway, which is particularly obvious in breast cancer cells (120).

#### 3.3.3 Metabolic regulation in cancer cells

LLPS plays a crucial role in the metabolic regulation of cancer cells. Cancer cells typically exhibit aberrant metabolic characteristics, such as enhanced glycolysis, altered lipid metabolism, dysregulated amino acid metabolism and so on (121). These abnormal metabolic phenotypes are closely linked to LLPS, as this physicochemical process can influence the aggregation state and activity of multiple metabolic enzymes.

On the one hand, LLPS can enhance metabolic efficiency and promote cancer cell proliferation by aggregating metabolic enzymes in membrane-less organelles. For example, in liver cancer cells, related metabolic enzymes such as the phosphofructokinase subunit Pfk2p are concentrated in membrane-less granules called glycolytic (G) bodies to improve hypoxia and meet high energy demands (122, 123). On the other hand, LLPS can participate in the regulation of substance metabolism. Studies have shown that LLPS affects the glucose metabolism of cancer cells, especially under glucose starvation conditions, through the formation and disintegration of SGs. When glucose is sufficient, LLPS promotes the formation of SGs, maintains high glycolytic activity, and supports rapid growth of cancer cells; When glucose starvation occurs, Sestrin2 mediates the disassembly and assembly of SGs, inhibits glycolysis, promotes metabolic adaptation (such as switching to oxidative phosphorylation), and helps cancer cells survive (14).

#### 3.3.4 Regulating tumor immunity

The formation of LLPS can reduce the infiltration of related immune cells, thereby inhibiting anti-cancer responses. The latest research shows that patient derived individual mutations in the NF2 FERM domain can convert NF2 into potent inhibitors of cGAS-STING signaling: activated IRF3 can directly bind to and induce mutant NF2 to form cell aggregates, which significantly reduces immune cell infiltration, especially CD4+and CD8+T lymphocytes, thereby eliminating STING induced anti-tumor immunity (124–126). The Eph receptor family is the largest receptor tyrosine kinase family, and EphA2, as a member of the Eph receptor family, exhibits LLPS characteristics in the occurrence and progression of various cancers, including colorectal cancer, liver cancer and breast cancer (127). The expression of EphA2 is not significantly correlated with common immune checkpoints such as PDCD1, CTLA4, and CD274, but it can enhance the infiltration of neutrophils, bone marrow dendritic cells, and macrophages, suggesting that EphA2 can regulate cancer development by affecting immune cell infiltration through LLPS (128).

The role of LLPS has also been reported in the B cell receptor (BCR) and T cell receptor (TCR) pathways. LLPS is closely related to the promotion of specific biochemical and signal transduction reactions mediated by TCR signaling pathways. For example, researchers revealed a new coagulation model related to TCR signaling: CD3 ϵ, a component of TCR, can form a condensate with Lck kinase through ion interaction, and its structure significantly promotes Lck mediated CD3 phosphorylation, thereby generating TCR signal amplification (129). Another study also revealed that when TCR phosphorylation is triggered, proteins including Lat and Lck spontaneously separate into liquid like clusters, promoting signal output from Jurkat T cells *in vitro* and in humans (130). In the BCR signaling pathway, a multivalent interaction occurs between the proline rich motif of SLP65 and the Src homology 3 (SH3) domain of CIN85, forming LLPS condensate (131). When the condensate approaches the plasma membrane, BCR phosphorylates SLP65, and downstream pathways are further triggered, including activation of the RAS pathway, mobilization of NF - κB, and calcium influx (132).

#### 3.3.5 Tumor progression and metastasis

The tumor microenvironment is a crucial factor in tumor development, providing necessary growth factors and nutrients for tumor cells and promoting their invasiveness (133). LLPS influences the formation of the tumor microenvironment by affecting the composition and structure of the extracellular matrix (ECM). Some ECM proteins, such as collagens and fibronectins, can form specific fibrillar networks through LLPS, providing scaffolds for cell adhesion (55). Studies have shown that collagen VI forms fibrils through an LLPS mechanism, and these fibrils can provide structural support and growth signals for tumor cells (134). Another study found that LLPS promotes the formation of fibronectin 1 fibrils, and this fibrillar structure influences the invasive ability of tumor cells (135).

Additionally, certain growth factors and ECM molecules, such as fibroblast growth factors, can interact with the ECM through LLPS, affecting their release and activity within the microenvironment. Tumor-associated fibroblasts remodel the tumor microenvironment by secreting these factors, promoting tumor development (136). The invasive and migratory abilities of tumor cells determine the extent of tumor development. Recent studies have shown that LLPS can regulate multiple key proteins associated with tumor invasion and migration, thereby affecting tumor progression (5). Some studies have found that certain transcription factors, such as Snail and Twist, can form LLPS droplets and regulate the expression of related genes like MMPs and VEGF, promoting the migratory ability of tumor cells (66). Furthermore, the cell-cell adhesion molecule E-cadherin can also form subcellular structures on the cell surface through the LLPS mechanism, participating in the regulation of adhesion and migratory abilities between tumor cells (137). In addition, LLPS can influence the distribution of ECM components within the tumor microenvironment, as shown by the ability of tumor-associated proteins, such as periostin, to promote collagen aggregation through LLPS, affecting the organization of the ECM and subsequently influencing the invasiveness of tumor cells (65).

## 4 Therapeutic potential of LLPS in cancer

LLPS may serve as a promising target for developing novel therapeutics for many devastating disorders, including neurodegenerative diseases, metabolic diseases, and autoimmune diseases (28, 138–140), offering new directions for cancer treatment. Cancer research has been a focal point in LLPS-related investigations, as modulating this process may offer promising therapeutic opportunities. Some studies have shown that intervening in the LLPS process of tumor cells can affect their metabolic pathways and exert anti-tumor effects. For example, certain small molecules can inhibit the LLPS of the nucleolar protein nucleolin, thereby disrupting tumor cell growth (141). Similarly, inhibiting the LLPS of the transcription factor FUS can suppress the proliferation and migration of cancer cells (142). Moreover, inhibitors targeting LLPS-related key proteins, such as transcription factors, are also under development. Interfering with the LLPS of Snail, a critical regulator of epithelial-mesenchymal transition, can inhibit the metastatic potential of tumor cells. As expected, there is evidence to suggest that small molecule drug therapy can effectively regulate LLPS, and we have summarized the inhibitors associated with LLPS in tumors and their mechanisms of action in **Table 3**.

**Table 3:** Inhibitors related to LLPS in tumors and their mechanisms of action.

Tumor	LLPS Inhibitors	Mechanism	Reference
Lung cancer	EVG	EVG disrupts the LLPS of SRC-1 and effectively inhibiting YAP oncogenic transcriptional activity.	(143)
	SHP099	SHP099 concurrently binds to the interface of the N-terminal SH2, C-terminal SH2, and protein tyrosine phosphatase domains, thus inhibiting SHP2 activity through an allosteric mechanism.	(144)
Breast Cancer	C108	C108 mediate the dissolution of condensates via modulation of SART3 mRNA regulation.	(145)
	Tamoxifen	Tamoxifen can reduce the formation of MED1 condensate in MYC oncogene, resulting in the expulsion of ER α from MED1 condensate, thus improving the drug resistance of breast cancer treatment.	(146)
Prostate Cancer	UT-143	UT-143 covalently and selectively binds to C406 and C327 in the AF-1 region, reversing LLPS and leading to chromatin condensation and dissociation of AR-V7 interactors, ultimately forming a transcriptional incompetent complex.	(147)
	LSD1-i	LSD-i inhibits prostate cancer by targeting multiple oncogenic pathways, including MYC signaling.	(148)
	ET516	ET516 destroys AR condensates, effectively inhibits AR transcriptional activity, and suppresses the proliferation and tumor growth of prostate cancer cells expressing AR resistant mutants.	(72)
Esophageal Cancer	TSA	TSA promotes epithelial mesenchymal transition (EMT) in ESCC cells by downregulating the epithelial marker E-cadherin and upregulating the mesenchymal markers β - catenin, vimentin, Slug, and PAI-1.	(149)
Osteosarcoma	GSK-J4	GSK-J4 can destroy CRC condensates, thereby inhibiting the proliferation of various osteosarcoma cell lines.	(150)
	Curcumin	Curcumin can weaken the sequestration of pyruvate kinase mediated by FUS aggregation and restore cellular metabolism, thereby increasing ATP levels.	(151)
Pancreatic Cancer	1,6-hexanediol	1,6-hexanediol disrupts protein-mediated abnormal LLPS and significantly reduce MYC expression.	(152)
	ZZW-115	ZZW-115 can affect the LLPS process of NUPR1 and prevent the presence of its related SGs, thereby triggering caspase 3 activation, DNA fragmentation, and the formation of apoptotic bodies, leading to cancer cell death.	(153)
Liver Cancer	JQ1	JQ1 can reduce the expression level of MYC, thereby effectively inhibiting mitochondrial glycolysis in HCC cells	(154)
Colorectal Cancer	Oxaliplatin	Oxaliplatin can alter the liquid-liquid mixing of nucleoli, modify nuclear RNA and proteins, leading to cell cycle arrest, Pol I-mediated transcriptional shutdown, and ultimately cell death.	(155)
	ACP-1n	ACP-1n inhibits BRD4 function in the nucleus of colorectal cancer cells by blocking LLPS to suppress SE-driven MYC expression.	(156)
	Cisplatin	Cisplatin can lead to a decrease in selectivity and progression of MED1 condensates, and inhibiting SE driven oncogene expression	(146)
	ALW-II-41-27	ALW-II-41-27 can disrupt the condensation formed by EphA2 on the cell membrane, thereby regulating iron cell content and immune cell infiltration.	(128)
Leukemia	HDACi	Mediating the separation of YY1 from HDAC1/3, leading to excessive LLPS status, thereby inhibiting the expression of METTL3 and the proliferation of AML cells.	(79)
	1,6-hexanediol	1,6-Hexanediol can eliminate the spheroids formed by EGFP-C/EBP α protein lines, thereby regulating the differentiation of acute myeloid leukemia cells.	(157)

In addition to small molecule inhibitors that inhibit the LLPS process, related studies have also shown that triggering LLPS can promote cancer cell death. For example, the new generation FSP1 inhibitor icFSP1 triggers subcellular repositioning and LLPS of FSP1 before ferroptosis and works synergistically with glutathione peroxidase 4 (GPX4) to inhibit tumor growth (158). This provides a reason for using FSP1 dependent LLPS as an effective anti-cancer therapy. In summary, targeting the LLPS process is a promising approach for developing new therapies to improve anti-cancer treatment. With the continued research in this field, the development of targeted LLPS inhibitors and their combined use with conventional therapies have great hope for improving the therapeutic effect and reducing the incidence rate and mortality associated with this devastating disease.

## 5 Conclusions and prospects

In this review, we have examined the multifaceted roles of LLPS in cell physiology and cancer biology. Our analysis highlights key insights into the involvement of LLPS in cancer, emphasizing its connection to various cancer hallmarks, including genomic instability, metabolic reprogramming, and immune evasion. By concentrating specific biomolecules while excluding others, LLPS creates unique microenvironments that can either promote or inhibit tumorigenic processes. Dysregulation of LLPS-mediated processes significantly contributes to cancer pathogenesis, as alterations in the LLPS properties of critical proteins, such as transcription factors and signaling molecules, can result in aberrant gene expression and signaling, driving cancer progression.

The therapeutic potential of LLPS in cancer treatment is particularly promising. Targeting the LLPS behavior of oncogenic proteins or disrupting cancer-promoting biomolecular condensates offers innovative strategies for anti-cancer therapies. Furthermore, the interplay between LLPS and post-translational modifications, particularly in cancer contexts, highlights the complexity of cellular regulation and the need for integrative research approaches. LLPS’s role in modulating the tumor microenvironment and immune responses also presents new opportunities for enhancing immunotherapy and overcoming treatment resistance.

Future research should focus on elucidating the molecular mechanisms of LLPS in cancer-specific contexts, developing advanced technologies for real-time monitoring of LLPS dynamics, and exploring LLPS-targeted therapies alongside conventional treatments. Investigating LLPS’s role in cancer stem cell maintenance and metastasis could address critical challenges in cancer therapy. Additionally, understanding the interaction between LLPS and epigenetic regulation may uncover novel targets for epigenetic therapies. By advancing our understanding of LLPS, we can pave the way for innovative diagnostic and therapeutic strategies in cancer treatment.

## Glossary

LLPS: liquid-liquid phase separation
IDR: intrinsically disordered region
SG: stress granule
LCD: low complexity domain
ssDNA: single-stranded DNA
SH2: src homology 2
FGFR2: fibroblast growth factor receptor 2
SHP2: SH2 domain-containing protein tyrosine phosphatase 2
PLCγ1: 1-phosphatidylinositol 4,5-bisphosphate phosphodiesterase gamma 1
PrLDs: prion-like domains
PRDX1: peroxiredoxin1
IncRNA: long non-coding RNA
HOXA9: homeobox A9
NHA9: NUP98–HOXA9
WBC: white blood cell
PTP: protein tyrosine phosphatase
SAM: Sterile Alpha Motif
PTM: post-translational modification
SMN: survival of motor neuron
RBD: RNA binding domain
RBP: RNA binding protein
eRNA: enhancer RNA
PolyQ: polyglutamine
HLBs: histone locus bodies
H1: histone 1
PRC1: poly-comb repressive complex 1
BP1: bromo-adjacent homology-plant homeodomain containing protein 1
KMT2D: lysine methyltransferase 2D
SENP1: Sentrin/SUMO specific protease 1
DDR: DNA damage response
DSBs: DNA double strand breaks
MRN: MRE11-RAD50-NBS1
MRNIP: MRN interacting proteins
53BP1: p53 binding protein
FUS: fused in sarcoma
miRISC: miRNA-induced silencing complex
HK: hexokinase
VDAC1: voltage- dependent anion channel I
BCR: B cell reporter
TCR: T cell reporter
ECM: extracellular matrix
GPX4: glutathione peroxidase 4
